# Attenuative effects of collagen peptide from milkfish (*Chanos chanos*) scales on ovariectomy‐induced osteoporosis

**DOI:** 10.1002/fsn3.3746

**Published:** 2023-10-08

**Authors:** Jiunn‐Jye Chuu, Jeng‐Wei Lu, Hung‐Ju Chang, You‐Hsiang Chu, Yi‐Jen Peng, Yi‐Jung Ho, Pei‐Hung Shen, Yu‐Shuan Cheng, Chia‐Hui Cheng, Yi‐Chien Liu, Chih‐Chien Wang

**Affiliations:** ^1^ Department of Biotechnology and Food Technology College of Engineering, Southern Taiwan University of Science Tainan Taiwan; ^2^ Biotech Research and Innovation Centre University of Copenhagen Copenhagen Denmark; ^3^ The Finsen Laboratory Rigshospitalet/National University Hospital, Faculty of Health and Medical Sciences, University of Copenhagen Copenhagen Denmark; ^4^ Department of Pathology Tri‐Service General Hospital, National Defense Medical Center Taipei Taiwan; ^5^ Graduate Institute of Life Sciences, National Defense Medical Center Taipei Taiwan; ^6^ School of Pharmacy, National Defense Medical Center Taipei Taiwan; ^7^ Department of Orthopedics Tri‐Service General Hospital, National Defense Medical Center Taipei Taiwan

**Keywords:** milkfish scales, osteoblast, osteoclast, osteoporosis, ovariectomized

## Abstract

Osteoporosis is characterized by low bone mass, bone microarchitecture disruption, and collagen loss, leading to increased fracture risk. In the current study, collagen peptides were extracted from milkfish scales (MS) to develop potential therapeutic candidates for osteoporosis. MS was used to synthesize a crude extract of fish scales (FS), collagen liquid (COL), and hydroxyapatite powder (HA). COL samples were further categorized according to the peptide size of total COL (0.1 mg/mL), COL < 1 kDa (0.1 mg/mL), COL: 1–10 kDa (0.1 mg/mL), and COL > 10 kDa (0.1 mg/mL) to determine it. Semi‐quantitative reverse transcription polymerase chain reaction (sqRT‐PCR) and immunofluorescence labeling were used to assess the expression levels of specific mRNA and proteins in vitro. For in vivo studies, mice ovariectomy (OVX)‐induced postmenopausal osteoporosis were developed, while the sham surgery (Sham) group was treated as a control. Collagen peptides (CP) from MS inhibited osteoclast differentiation in RAW264.7 cells following an insult with nuclear factor kappa‐B ligand (RANKL). CP also enhanced osteoblast proliferation in MG‐63 cells, possibly through downregulating NFATc1 and TRAP mRNA expression and upregulating ALP and OPG mRNA levels. Furthermore, COL1 kDa also inhibited bone density loss in osteoporotic mice. Taken together, CP may reduce RANKL‐induced osteoclast activity while promoting osteoblast synthesis, and therefore may act as a potential therapeutic agent for the prevention and control of osteoporosis.

## Graphical abstract

Collagen peptide from milkfish (*Chanos chanos*) may reduce RANKL‐induced osteoclast activity while promoting osteoblast synthesis, and therefore may act as a potential therapeutic agent for the prevention and control of osteoporosis.

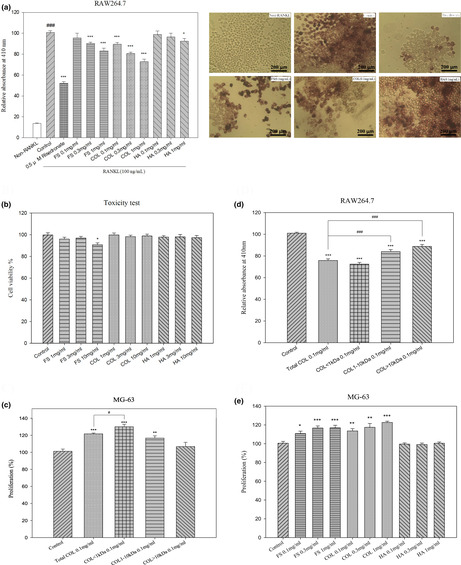

## 1 INTRODUCTION

Osteoporosis is characterized by low bone mass and the structural deterioration of bone tissue, with an increased risk of fracture. The World Health Organization (WHO) has identified osteoporosis as a serious health issue affecting millions of people worldwide. The incidence of osteoporosis increases with age and occurs most frequently in postmenopausal women (Pignolo et al., 2021), suggesting a link with estrogen deficiency. As per the International Osteoporosis Foundation (IOF), 158 million adults over 50 years of age and older are at enhanced risk of osteoporotic fracture globally, which is anticipated to double by 2040 (Hsu et al., 2022). In Taiwan, the average incurred cost of each fracture case exceeds 80,000 NTD for the initial therapy (Hwang et al., 2014). To date, though various therapies are being explored, hormone replacement therapy (HRT) is widely recommended for postmenopausal women seeking to prevent bone loss and fractures; however, it has been shown to exert estrogen‐like adverse effects, such as breast cancer, stroke, and heart attack (Prelevic et al., 2005). The efficacious methods of osteoporosis include the inhibition of osteoclast‐mediated bone resorption and increasing the formation of new bone by osteoblasts. Recent developments in osteoporosis treatments indicate their focus on preventing bone resorption, and only a few of them aim to promote bone growth. A previous study reported that cell‐to‐cell communication molecules involved in bone remodeling (e.g., nuclear factor kappa‐B ligand (RANKL) produced by osteoblasts) play an essential role in the structural deterioration of bone tissue (Kim & Kim, 2020). This indicates that a balance between osteoblasts and osteoclasts is crucial to bone health.

As per recent clinical studies, collagen‐associated bioproducts have been observed to ameliorate joint symptoms and arthralgia (Clark et al., 2008). Collagen is the most important constituent of protein in milkfish (MS) (*Chanos chanos*), a marine aquaculture species in Taiwan (Hu et al., 2015), which imparts physical protection to tissues [1]. Annually, a large amount of fish is harvested, and around 50% of protein‐rich fish processing byproducts are thrown out or employed as fishmeal and animal feed without any effort to recuperate the essential nutrients (Kristinsson & Rasco, 2000). The use of collagen from marine life is rapidly increasing due to its biocompatibility, bioavailability, low risk of disease transmission, and low molecular weights (Jafari et al., 2020). Collagen is the most common structural protein in the extracellular matrix of connective tissue, skin, bones, ligaments, tendons, and cartilage. Collagen is generally used in tissue engineering as an osteogenic and bone‐filling material, a hemostatic agent, and burn/wound dressing (Dong & Lv, 2016; Jafari et al., 2020). Marine collagen from vertebrates or invertebrates provides bioavailability superior to that of bovine or porcine collagen, due to its low molecular weight, small particle size, high absorbency, and ease of circulation in the bloodstream (Dong & Lv, 2016). In terms of amino acid content and biocompatibility, marine‐based collagens are comparable to bovine and pig collagen (Carvalho et al., 2018; Jafari et al., 2020). Fish collagen can be made from a variety of fish by‐products, including bones, scales, and skin, i.e., waste from fish processing plants (Blanco et al., 2017; Jafari et al., 2020).

Collagen peptides (CP) are rich in amino acids, including glycine, proline, and hydroxyproline. These amino acids all play an important role in the construction of joint cartilage, may also have anti‐inflammatory, anti‐aging effects, wound healing effects, neuroprotective effects, and antioxidant effects, and are presumed to act as signaling molecules (Asserin et al., 2015; Cheung et al., 2015; Ghorpade et al., 2016; Mobasheri et al., 2021; Paul et al., 2019). An oral type II collagen supplement was found to reduce joint swelling in rheumatoid arthritis (RA) (Trentham et al., 1993). In addition, fish collagen peptides have also been found to reduce the production of pro‐inflammatory cytokines and prevent cisplatin‐induced cytotoxicity and oxidative damage in thymic epithelial cells (Ding et al., 2003; Song et al., 2022).

There is growing interest in the treatment of bone tissue using collagen extracted from fish scales. Collagen is present in connective tissues such as skin, bones, tendons, and cartilages. It is made up of three polypeptide chains with a triple‐helix molecular structure and three repeating residues of (Gly‐Pro‐Hyp, GPH)n (Xu et al., 2021). The scales from teleost fish are a good model by which to study osteoclast activity in age‐related bone resorption and osteoblast activity in the formation of new bone (Kitamura et al., 2013). Milkfish scale collagen peptides (MSCP) have been shown to possess antioxidant and anti‐inflammatory properties (Chen et al., 2018). In the current study, MSCP was extracted using the hollow fiber column‐soluble collagen technique and specifically aimed to investigate the effects of MSCP on osteoblast and osteoclast under osteoporosis environment.

## 2 MATERIALS AND METHODS

### 2.1 Chemicals and reagents

Thiazolyl blue tetrazolium bromide and risedronate were purchased from Sigma‐Aldrich Inc. The TRAP staining kit was purchased from Cosmo Bio Co. Dulbecco's Modified Eagle Medium (DMEM), hematoxylin and eosin (H&E), 4′,6‐Diamidino‐2‐Phenylindole (DAPI), as well as p38 MAPK beta and AP1/JUN/RUNX2/OCN antibodies were purchased from Thermo Fisher Scientific Inc. Integrated DNA Technologies provided the primers (NFATc1, TRAP, GAPDH (Mouse), ALP, OPG, and GAPDH (Human)). AMRESCO provided a phenol‐free total RNA purification kit.

### 2.2 Extraction of fish scale crude extract, collagen peptides, and hydroxyapatite

The heated fish scales were pulverized into small pieces by disperser, then subjected to hydrolysis under 1% protease N for 2.5 h, and then 0.5% flavourzyme (Novozymes) for another 0.5 . The hydrolysates were heated in a boiling water bath with stirring for 10 min to inactivate the enzyme, and then the hydrolysates were centrifuged at 12,000 ***g*** for 20 min according to the previous protocol (Chai et al., 2010). We then performed freezing‐drying to separate the precipitate (hydroxyapatite powder; HA) from the supernatant (collagen liquid; COL) (Kingmech, FD 20L‐6S). After dissolving the HA powder in double‐distilled water, a hollow fiber column (Spectrum Labs) was used to extract peptides of various sizes, which were subsequently freeze‐dried to form a powder. The peptides in the powder presented the following size distribution: total COL (0.1 mg/mL), COL < 1 kDa (0.1 mg/mL), COL: 1–10 kDa (0.1 mg/mL), and COL > 10 kDa (0.1 mg/mL). Finally, the peptides were prepared by redissolving in sterilized double‐distilled water and filtered through a 0.22 μm syringe filter (Millipore).

### 2.3 Inhibition or promotion of osteoclast differentiation and proliferation

Mouse BALB/c macrophages transformed by the Abelson murine leukemia virus (RAW264.7) were obtained from the Food Industry Research and Development Institute (Hsinchu, Taiwan). RAW264.7 cells were supplemented using DMEM medium with 10% Fetal Bovine Serum (FBS), 2 mM L‐glutamine, 1.5 g/L sodium bicarbonate, 4.5 g/L glucose, 10 mM N‐2‐hydroxyethylpiperazine‐N‐2‐ethane sulfonic acid (HEPES), and 1.0 mM sodium pyruvate. When the RAW 264.7 cells reached a growth density of 1 × 10^3^ in a 96‐well plate, the medium was replaced with an α‐minimum essential medium (α‐MEM). After incubation for 24 h, the cells were cultured with RANKL (5 ng/mL) in conjunction with risedronate (5 μg/mL), FS (0.1 and 0.3 mg/mL), COL (0.1 and 0.3 mg/mL), or HA (0.1 and 0.3 mg/mL) for 4 days, whereupon TRAP staining was performed. Briefly, cells were fixed in 10% (v/v) formalin for 10 min, washed in 95%(v/v) ethanol, and then incubated in 0.1 mL of phosphatase substrate (3.7 mm p‐nitrophenyl phosphate in 50 mm citrate buffer, pH 4.6) in the presence of 10 mm sodium tartrate at 25°C for 30 min to evaluate TRAP activity. The solution from each well was transferred to a tube containing 0.1 mL of 0.1 n NaOH after incubation, and the absorbance at 410 nm was measured. The cells were stained for TRAP with 0.1 mg/mL naphthol AS‐MX phosphate and 0.6 mg/mL fast red violet LB salt in 0.1 m sodium acetate buffer, pH 5.0, including 50 mm sodium tartrate, after the TRAP activity assay. We counted all TRAP‐positive cells with three or more nuclei (Hirotani et al., 2004). Note that the cell culture medium was changed every 2 days. MG‐63 cells were seeded in 96‐well plates at a concentration of 1 × 10^4^ cells per well with the addition of 3‐(4,5‐Dimethylthiazol‐2‐yl)‐2,5‐diphenyltetrazolium bromide (MTT) solution, followed by incubation at 37°C for 24 h. The supernatant was then removed, and the wells were rinsed three times using PBS. After the wells were dried, 200 μL of DMSO was added. The plates were then gently shaken to facilitate the dissolution of the formazan crystals. An enzyme‐linked immunosorbent assay (ELISA) reader was used to measure absorbance at a wavelength of 570 nm.

### 2.4 The mRNA isolation and semi‐quantitative reverse transcription polymerase chain reaction (sqRT‐PCR)

RNA was isolated from RAW264.7 and MG‐63 cells that had been administered total COL (0.1 mg/mL), COL < 1 kDa (0.1 mg/mL), COL: 1–10 kDa (0.1 mg/mL), or COL > 10 kDa (0.1 mg/mL) using a phenol‐free total RNA purification kit. cDNA was synthesized using an iScript reverse transcriptase kit (Bio‐Rad Laboratories), and the cDNA templates were amplified using sqRT‐PCR with 1 U of Thermus Aquaticus (Taq) polymerase (AmpliTaq; Perkin‐Elmer). One microliter of cDNA was amplified via PCR, which involved 1 cycle at 95°C for 3 min, 25 cycles at 94°C for 1 min, 65°C for 30 s, and 72°C for 1 min, followed by incubation at 72°C for an additional 7 min for the completion of the synthesis. The following primers were used to assess gene expression in the cDNA: NFATc1 (Plus: 5′‐TGG GAG ATG GAA GCA AAG AC‐3′/Minus: 5′‐TGG GAG ATG GAA GCA AAG AC‐3′); TRAP (Plus: 5′‐AGA CCC AAT GCC ACC‐3′/Minus: 5′‐GGA CCT CCA AGT TCT TAT C‐3′); GAPDH (Mouse) (Plus: 5′‐AAG CCC ATC ACC ATC TTC CAG‐3′/Minus: 5′‐AAG CCC ATC ACC ATC TTC CAG‐3′); ALP (Plus: 5′‐CCA ACT CTT TTG TGC CAG AGA‐3′/Minus: 5′‐CCA ACT CTT TTG TGC CAG AGA‐3′); OPG (Plus: 5′‐ACC CAG AAA CTG GTC ATC AGC‐3′/Minus: 5′‐CTG CAA TAC ACA CAC TCA TCA CT‐3′); and GAPDH (Human) (Plus: 5′‐AGG TCG GTG TGA ACG GAT TTG‐3′/Minus: 5′‐AGG TCG GTG TGA ACG GAT TTG‐3′). The band strengths were analyzed using an Instant Imager (Camberra‐Packard) to quantify the RAW 264.7 trap/GAPDH and NFATc1/GAPDH ratios at 2 and 4 days, as well as the MG‐63 ALP/GAPDH and OPG/GAPDH ratios at 6, 12, and 24 h. Quantification of the sqRT‐PCR data using Image J software (NIH) (Stolberg‐Stolberg et al., 2022; Vandesompele et al., 2002).

### 2.5 Immunofluorescence staining and quantification

RAW 264.7 cells and MG‐63 cells were treated with total COL (0.1 mg/mL), COL < 1 kDa (0.1 mg/mL), COL: 1–10 kDa (0.1 mg/mL), or COL > 10 kDa (0.1 mg/mL) for 24 h, followed by rinsing in ice‐cold phosphate‐buffered saline (PBS) and then fixed using PBS with 4% paraformaldehyde at 37°C for 30 min. The cells were then rinsed twice using ice‐cold PBS before being incubated in 0.25% Triton X‐100 in 0.1% bovine serum albumin (BSA) at 4°C for 5 min. To prevent non‐specific binding, the cells were washed twice using ice‐cold PBS and incubated in PBS containing 0.1% BSA at room temperature for 1 h. The cells were then incubated overnight at 4°C in PBS containing 0.1% BSA with antibodies diluted 1:200 against p38 (Thermo Fisher Scientific), AP‐1 (Thermo Fisher Scientific), RUNX2 (Thermo Fisher Scientific), or OCN (Thermo Fisher Scientific). The cells were washed using PBS three times and then incubated at room temperature in PBS containing 0.1% BSA with anti‐rabbit FITC (1:500) for 1 h. After three PBS washes, the nuclei were counter‐stained in the dark using 5 g/mL DAPI (Sigma‐Aldrich) for 10 min, before being photographed using a fluorescence microscope (Olympus IX81). Immunofluorescence was performed in accordance with the methods outlined in a previous study. Quantification of the Immunofluorescence staining data using Image J software (NIH). Quantification of the percentage of positive cells for the all fields using the Image J software. The quantification of the data was independently calculated by two blinded, experienced pathologists according to previous methods (Liu et al., 2019).

### 2.6 Simulated postmenopausal animal model and alkaline phosphatase (ALP) activity

Female BALB mice (6 weeks of age) from the Institute of Cancer Research (ICR) were purchased from BioLASCO Taiwan Co., Ltd. All animal experiments were approved by the Accreditation of Laboratory Animal Care in accordance with the Institutional Animal Care and Use Committee of the Animal Research Committee (IACUC) of the Southern Taiwan University of Science and Technology (approval no. STUST‐IACUC‐106‐3). All experiments were conducted in accordance with the Guide for the Care and Use of Laboratory Animals, as outlined by the National Institutes of Health. Prior to surgical insult, the mice were acclimated to 12/12‐h light–dark cycle conditions in a housing facility for a period of 1 week. Mice were subjected to ovariectomy (OVX) through the back or sham surgery (Sham), which involved pulling out the ovary and then replacing it using 5‐0 sutures purchased from Johnson & Johnson Inc. (USA). Eight weeks after simulated postmenopausal surgery, a 4‐week treatment trial was conducted. The study groups included the following: negative control group (Saline), positive control group (Risedronate, 30 μg/kg/day), high‐dose FS crude extract group (300 mg/kg/day), COL low‐dose group (100 mg/kg/day), COL high‐dose group (300 mg/kg/day), COL < 1 kDa peptide high‐dose group (300 mg/kg/day), COL: 1–10 kDa peptide high‐dose group (300 mg/kg/day), and COL > 10 kDa peptide high‐dose group (300 mg/kg/day). The effects of celecoxib (30 mg/kg) were relative to the treatment group (positive control). Throughout the experiments, blood serum metabolic enzymes were quantified using an ELISA test, including ALP (IU/L).

### 2.7 Histopathological examination

The harvested knees of the mice were fixed in 4% paraformaldehyde, decalcified in 9% formic acid for 3–5 days, and embedded in paraffin. Using an RM2135 microtome (Leica Microsystems Inc.), formalin‐fixed tissues were cut into 5‐μm‐thick slices, placed on silane‐coated slides, and submerged in tris‐buffered saline (pH 7.4). The samples were then dried overnight at 37°C, rehydrated using graded ethanol solutions, and maintained at room temperature. Serial H&E staining (Sakura Finetek) of OVX cartilage revealed severe cartilage breakdown and calcification, as indicated by the pale blue hue. We used the 14‐point Mankin score to evaluate OVX cartilage. Note that the consistency with which H&E staining is used to diagnose cartilage lesions is a crucial restraint on the validity of scoring. When stained with hematoxylin, basophilic compounds in the nucleus and cytoplasm turn blue‐purple. The attachment of eosin to collagen fibers turns them red, thereby making it possible to assess the structural integrity of the extracellular matrix in cartilaginous tissue. The histological categorization of cartilage degradation during OVX development involved staining the samples with safranin‐O/fast green and then analyzing the slides using a Motic BA 400 microscope in conjunction with Motic Advance 3.0 software (Motic Co.).

### 2.8 Statistical analysis

All experiments were conducted in triplicate. One‐way anovas and Tukey's post‐hoc tests were used to compare mean values across groups using Sigma Plot 10.0 software (SPSS Inc.). *p < .05, **p < .01, ***p  < .001 and #*p* < .05, ##*p* < .01, ###*p* < .001 respectively.

## 3 RESULTS

### 3.1 Low‐MW collagen peptide abrogated RANKL‐induced osteoclast formation and promoted the proliferation of osteoblasts

**FIGURE 1:**
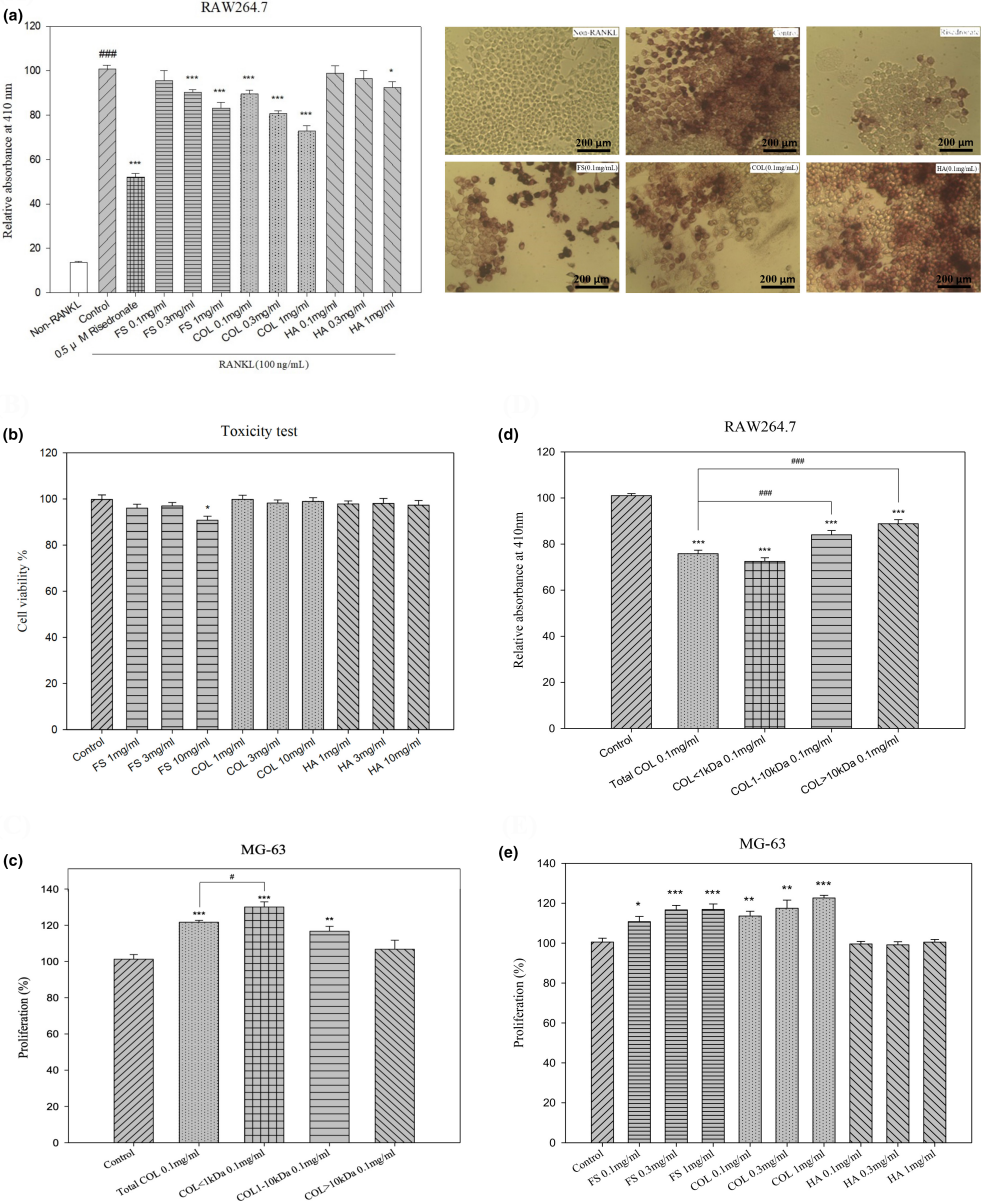
In vitro cell pharmacological test. (a) Test results and representative microscopic images showing the effects of extracts from milkfish scales on osteoclast (RAW264.7) differentiation. Control, risedronate (30 μg/mL) as well as FS, COL, and HA (0.1, 0.3, and 1 mg/mL, respectively); Scale bar = 200 μm. (b) Effects of FS, COL, or HA on osteoclast (RAW264.7) toxicity, where the concentration is 1, 3, or 10 mg/mL, respectively. (c) Test results showing the effects of milkfish scale collagen peptide on osteoblast (MG‐63) proliferation. Control, total COL, COL: 1–10 kDa, and COL > 10 kDa (0.1 mg/mL). (d) Effects of milkfish scale CP on osteoclast differentiation (RAW264.7), where the concentration of control, total COL, COL < 1 kDa, COL: 1–10 kDa, and COL > 10 kDa is 0.1 mg/mL. (e) Experiment on the promotion of osteoblast proliferation by various extracts from milkfish scales. The concentrations of control as well as FS, COL, and HA are 0.1, 0.3, and 1 mg/ ml, respectively. The values in the figure are expressed as the mean ± SE(*n* = 6). The significance was indicated as follows: *p < .05, **p < .01, and ***p < .001 compared with the control groups or #*p* < .05, ##*p* < .01, and ###*p* < .001 compared with non‐RANKL groups or total COL groups.

First, to investigate the effect of low‐MW collagen peptide (LCP) on RANKL‐induced osteoclastogenesis, we examined the production of osteoclasts in RANKL‐treated RAW264.7 cells cultured with various concentrations (0.1, 0.3, or 1 mg/mL, respectively) of fish scales (FS), collagen liquid (COL), or hydroxyapatite powder (HA). Treatment with three different concentrations of FS, COL, or HA was shown to reduce the number of RANKL‐induced osteoclasts in a dose‐dependent manner (*p* < .05, *p* < .01, and *p* < .001, respectively) (Figure 1a). Second, a toxicity test was used to evaluate cell viability for FS, COL, or HA with three concentrations (1, 3, or 10 mg/mL, respectively) (Figure 1b). Third, this prompted us to perform a more in‐depth study of FS collagen peptides, which involved repeating the in vitro cell pharmacological test using collagen peptides at three sizes of <1, 1–10, and >10 kDa (0.1 mg/mL, respectively). While investigating inhibition of osteoclast differentiation (*p* < .001), the <1 kDa collagen peptide group presented the most pronounced pharmacological effects, with inhibitory effects of total COL exceeding those of 1–10 kDa and >10 kDa peptides. Based on these findings, we determined that LCP (1 kDa) reduced RANKL‐induced osteoclastogenesis. We also performed osteoblast proliferation experiments using MG‐63 cells (Figure 1c). In addition, treatment with collagen peptides was also shown to increase the percentage of proliferating in Total COL, COL < 1 kDa, or COL: 1–10 kDa (0.1 mg/mL, respectively) compared with the control or total COL groups (*p* < .05, *p* < .01, and *p* < .001, respectively) (Figure 1d). Finally, treatment with FS, COL, or HA was shown to increase the percentage of proliferating in FS and COL (0.1, 0.3, and 1 mg/mL, respectively) compared with the control group (*p* < .05, *p* < .01, and *p* < .001, respectively) (Figure 1e).

### 3.2 LCP suppressed TRAP and NFATc1 mRNA expression in RANKL‐induced RAW264.7 macrophage cells in a time‐dependent manner

**FIGURE 2:**
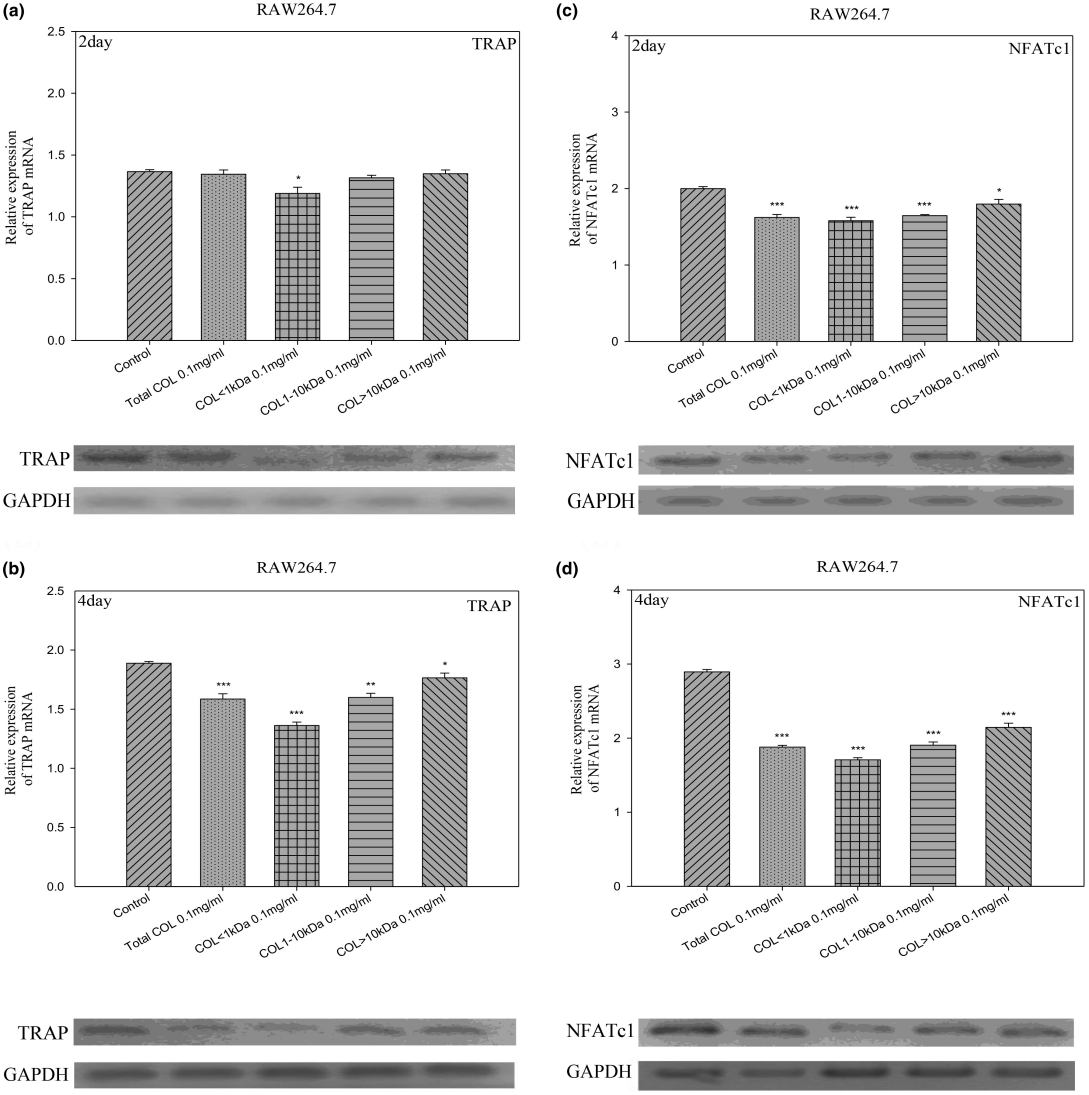
The mRNA expression levels of the NFATc1 and TRAP genes in osteoclasts. (a–d) Inhibition of osteoclast (RAW264.7) differentiation as a function of peptide size, as indicated by the expression of TRAP or NFATc1 genes. COL < 1 kDa (0.1 mg/mL) had the most striking impact on downregulating osteoclast gene expression at 2 and 4 days. The concentrations of control, total COL, COL < 1 kDa, COL: 1–10 kDa, and COL > 10 kDa (0.1 mg/mL). Data are expressed as mean ± SE (*n* = 3). *p < .05, **p < .01, and ***p < .001, compared with the control groups.

We explored the effect of LCP on the differentiation of RAW264.7 macrophage cells in the absence or presence of LCP by measuring the mRNA expression levels of RANKL‐induced osteoclast‐associated genes. NFATc1 is a downstream transcription factor in the RANKL/receptor activator of NF‐κB (RANK) signal pathway, and as a key molecule of osteoclastogenesis, NFATc1 induces a series of osteoclast‐specific genes, including TRAP. NFATc1 and TRAP are good markers of important transcription factors in the process of osteoclast differentiation and function (Asagiri et al., 2005; Du et al., 2020; Kong et al., 2013; Zhao et al., 2010). TRAP and NFATc1 mRNA expression during osteoclastogenesis was both suppressed in a dose‐dependent manner following administration with collagen peptides at various concentrations (total COL as well as COL < 1 kDa, 1–10 kDa, and >10 kDa) for 2 or 4 days (*p* < .05, *p* < .01, and *p* < .001, respectively) (Figure 2). These results support the assertion that LCP inhibits osteoclastogenesis and bone resorption.

### 3.3 LCP promoted cell differentiation in cultured MG‐63 osteoblast cells through the upregulation of ALP and OPG mRNA expression

Alkaline phosphatase is a biochemical marker of osteoblasts, as the ALP enzyme is secreted during osteoblast differentiation. A wide range of cell signals, hormones, and growth factors are regulated by RANKL as well as its receptors, RANK and OPG. RANKL binding to RANK promotes osteoclast differentiation and functionality (Yazid et al., 2010). OPG is also a good marker of differentiation in the osteoclast (Kwan Tat et al., 2008).

**FIGURE 3:**
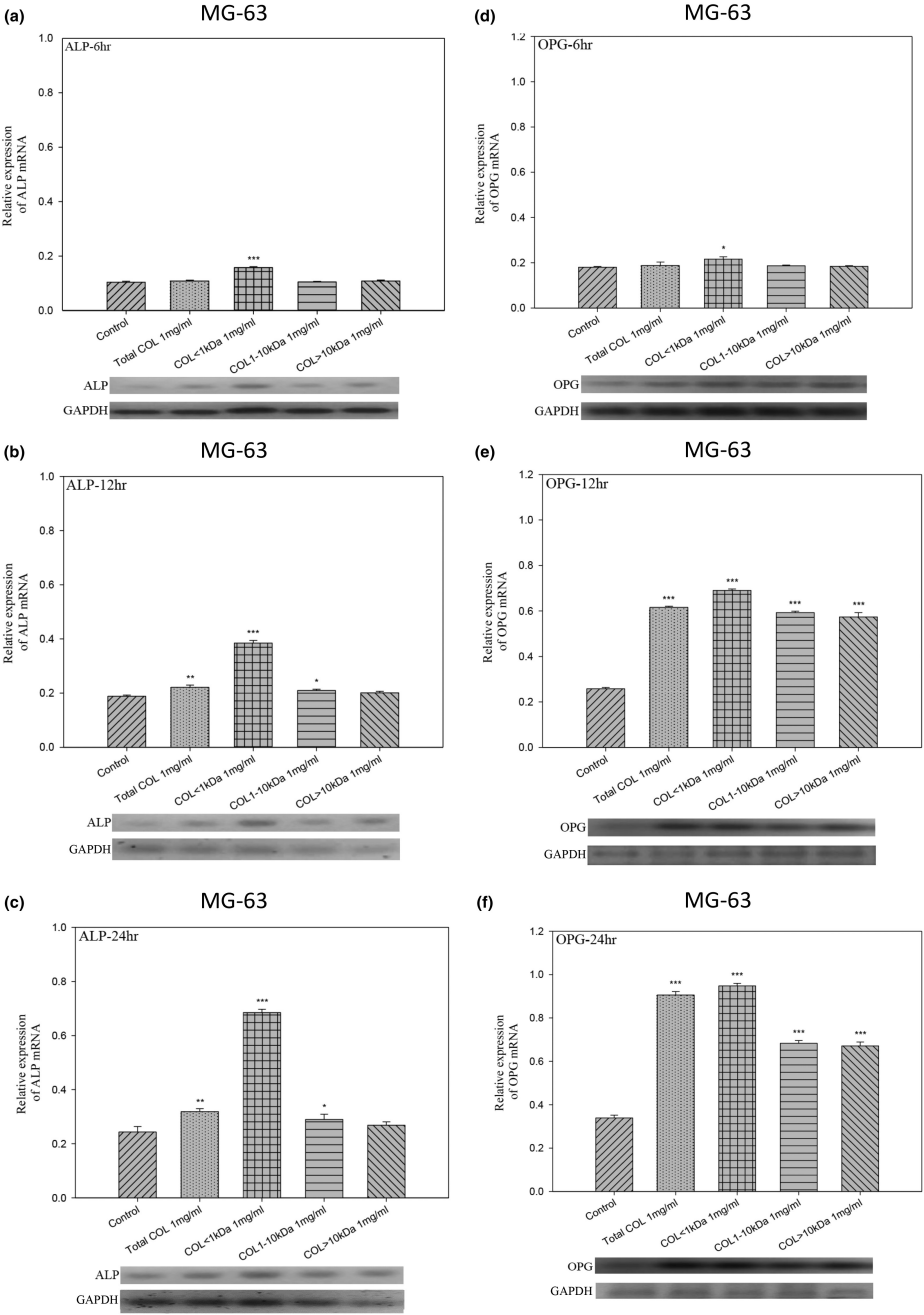
The mRNA expression levels of the OPG and ALP genes in osteoblasts. (a–f) MG‐63 osteoblasts were subjected to CP of various sizes for 6, 12, or 24 h to observe their effect on ALP and/or OPG gene expression. The concentrations of control, total COL, COL < 1 kDa, COL: 1–10 kDa, and COL > 10 kDa was 0.1 mg/mL. Normalized gene expression levels are expressed as ratios of the number of mRNA copies of specific genes versus the number of GAPDH cDNA copies. Treatment with CP (<1 kDa) showed the most pronounced effect on downregulating osteoblast gene expression at 6, 12, and 24 h. Data are expressed as mean ± SE (*n* = 3). *p < .05, **p < .01, and ***p < .001, compared with control groups.

Following 6‐, 12‐, or 24‐h treatment with collagen peptide at various concentrations (total COL as well as COL < 1 kDa, 1–10 kDa, and >10 kDa), ALP and OPG tests were performed to identify the optimum dose of LCP by which to promote MG‐63 osteoblast development. Treatment with 1 mg/mL COL < 1 kDa significantly enhanced ALP and OPG activity; however, lower doses were ineffective. ALP activity under treatment with COL < 1 kDa or OPG peaked at roughly 24 h (*p* < .05, *p* < .01, and *p* < .001, respectively) (Figure 3).

### 3.4 Inhibitory effects of LCP on signaling on the p38/AP‐1 pathway during RANKL‐induced osteoclast development

**FIGURE 4:**
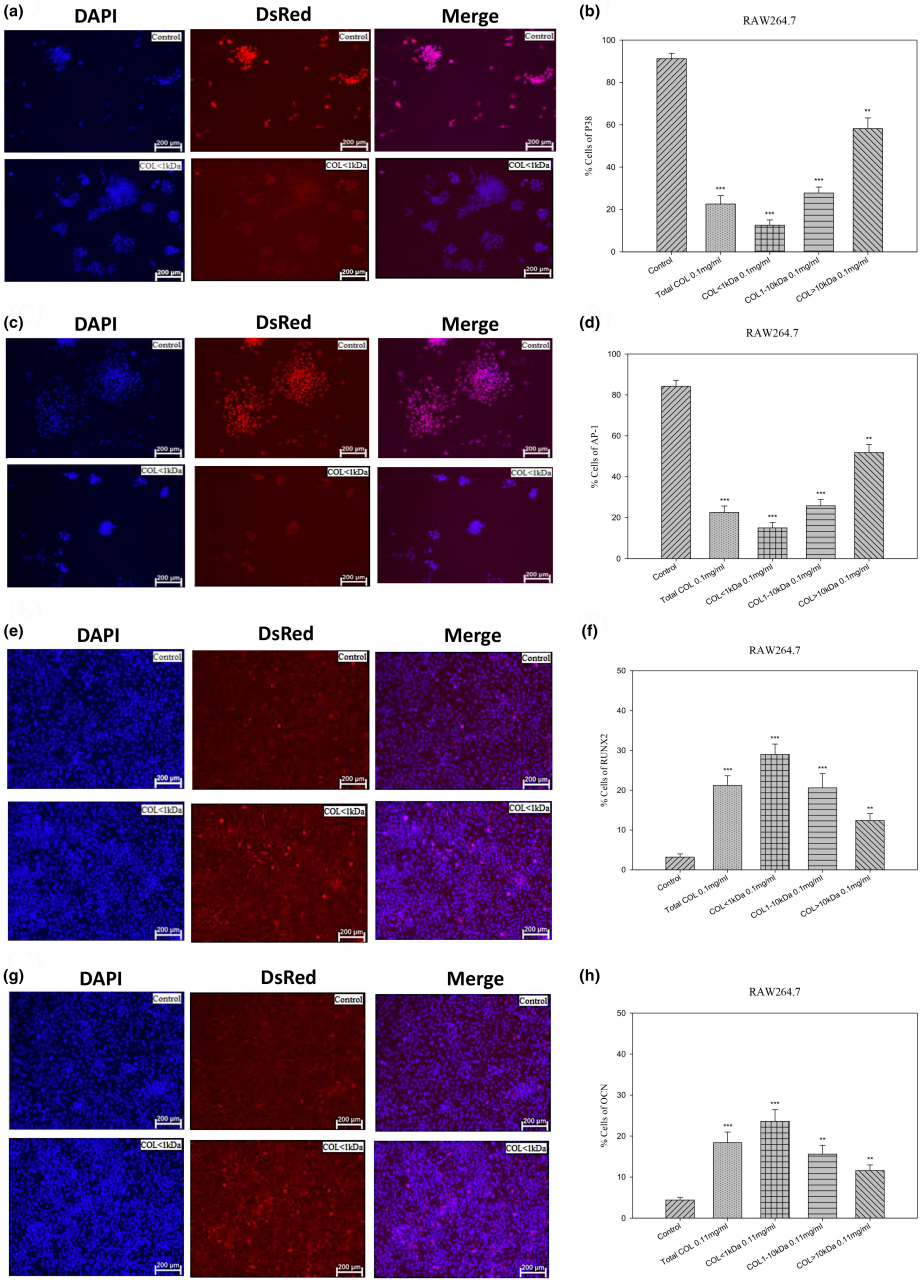
Osteoclast and osteoclastogenesis immunofluorescence staining assay. (a, c, e, and g) Different sizes of milkfish scale CP were used to assess their impact on inhibition of osteoclasts differentiation (RAW264.7) by calculating the expression of P38, AP‐1, RUNX2, and OCN proteins with their respective quantifications. The concentration of control, total COL, COL < 1 kDa, COL: 1–10 kDa, and COL > 10 kDa is 0.1 mg/mL). Immunofluorescence staining of P38, AP‐1, RUNX2, or OCN as well as quantification of the percentage of positive cells (b, d, f, and h): Extranuclear and intranuclear osteoclast gene expression revealed that COL < 1 kDa (0.1 mg/mL) had the most pronounced effect on the downregulation or upregulation of osteoclast gene expression. The data are expressed as mean ± SE (*n* = 5). *p < .05, **p < .01, and ***p < .001, compared with the control groups. Scale bar = 200 μm.

In this in vitro cell model, RAW264.7 cells were treated with collagen peptide at various concentrations (total CO as well as COL < 1 kDa, 1–10 kDa, and >10 kDa). We detected the expression of p38 and AP‐1. Our immunofluorescence results revealed a significantly sharp decline in p38 (*p* < .01), and AP‐1 activity (*p* < .001, with in COL < 1 kDa‐treated group, implying its presenting the most pronounced downregulation of osteoclast gene expression (Figure 4a–d).

### 3.5 LCP increased osteogenic differentiation in MG‐63 cells through the activation of RUNX2/OCN signaling pathways

In this in vitro cell model, MG‐63 cells were treated with collagen peptide (total COL as well as COL < 1 kDa, 1–10 kDa, and >10 kDa). We then detected the expression of RUNX2 and OCN. Our immunofluorescence analysis revealed RUNX2 and OCN activity, with COL < 1 kDa presenting the most pronounced upregulation of osteoclast gene expression (*p* < .01 and *p* < .001, respectively) (Figure 4e–h).

### 3.6 Improving the impact of LCP on serum ALP levels in ovariectomized‐induced osteoporosis mice

**FIGURE 5:**
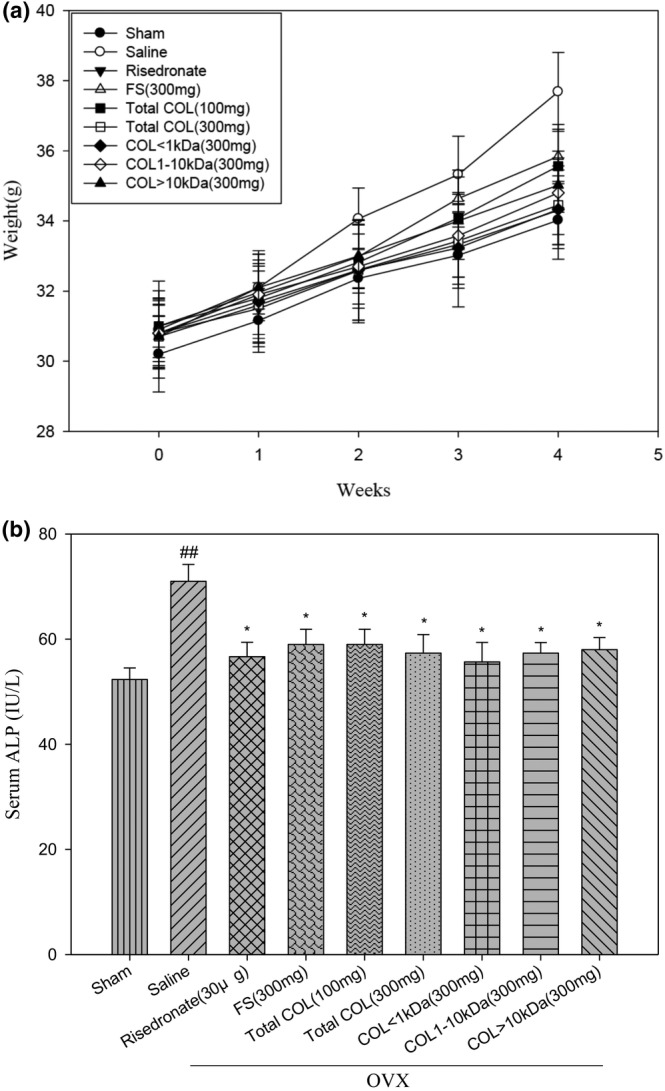
(a) Body weight of mice in the various treatment groups. The number of samples, *n* = 5. (b) ALP serum levels in sham, saline, risedronate groups (30 μg/mL), FS group (300 mg/mL), total COL group (100 and 300 mg), COL <1 kDa group (300 mg/mL), COL: 1–10 kDa group (300 mg/mL), and COL > 10 kDa group (300 mg/mL). The ALP content in the blood of mice in the saline group and the sham group was highest, whereas that of the other experimental groups all decreased, which was comparable to the ALP content in the sham group without ovariectomy. Data are represented as mean ± SE (*n* = 3). *p < .05, **p < .01, and ***p < .001 compared with the saline groups or #*p* < .05, ##*p* < .01, and ###*p* < .001 compared with the sham group.

The in vivo results revealed that no significant change in body weight of the experimental treatment groups of mice was found when compared with the sham or saline groups throughout the treatment period. (Figure 5a). We then evaluated serum ALP levels (an indicator of bone production) in ovariectomized mice, which were 1.37‐fold higher in the saline group than in the sham group (*p* < .01). Results show that all treatment groups of risedronate FS, total COL as well as COL < 1 kDa, 1–10 kDa, and >10 kDa (30 μg, 100 or 300 mg/mL, respectively) significantly decrease serum ALP levels compared with the saline group, respectively (*p* < .05) (Figure 5b). In addition, the risedronate (bisphosphonate) was shown to slow disease progression as measured by cartilage lesion size, severity, and osteophyte size, with a maximal effect of disease suppression of 30%–40%, where risedronate was considered as a positive control (Iwamoto et al., 2010).

### 3.7 Effect of LCP on thickness and number of trabecular bone formations in ovariectomized‐induced osteoporosis mice

**FIGURE 6:**
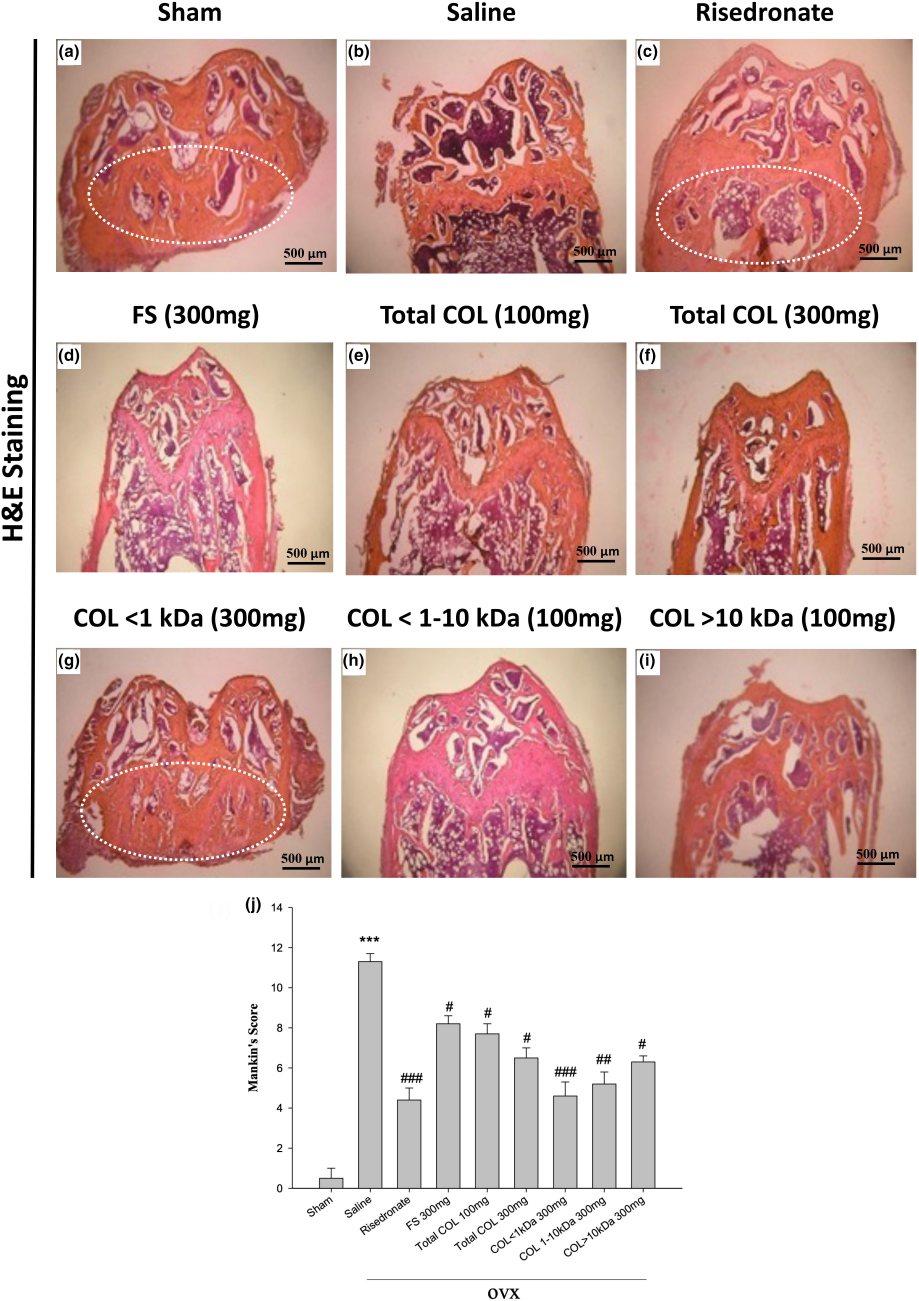
Histomorphological results from the femur of OVX mice. (a–i) At 28 days after surgery, the animals were sacrificed for histological analysis of the femur. Note that treatment involved FS (300 mg/kg/day), total COL (100 and 300 mg/kg/day), COL < 1 kDa (300 mg/kg/day), COL: 1–10 kDa (300 mg/kg/day), COL > 10 kDa (300 mg/kg/day), and risedronate (30 μg/kg/day) in OVX mice: (a) Femoral tissue in the sham‐operated group (white rectangle frame) remained relatively intact, and bone density was well preserved compared with (b) the saline group (OVX). We found better bone density based on veterinary pathologist grading. As shown in the white circle, the bone tissue was relatively tight. (a) In the white circle, in addition to the sham group without ovariectomy, (c) there is also a risedronate group, and (g) COL < 1 kDa (300 mg/kg/day), which revealed higher bone density and indicated enhanced therapeutic effect compared with the saline group. The rest of the experimental groups showed slight improvement. Scale bar = 500 μm. (j) Quantification of scores for all fields of stained sections in each group (*n* = 5). ***p < .001, ^#^ *p* < .05, ^##^ *p* < .01, and ^###^ *p* < .001, respectively.

We investigated the positive effects of LCP extracts on the trabecular bone of the proximal femur in ovariectomized‐induced OVX mice by histologic staining of bone sections with H&E, followed by optical imaging. In early OVX, changes in OA were found in our mice model. H&E staining (white circle) indicated bone saturation at the femoral joint, similar to healthy mice in the sham‐operated control group (Figure 6a). The saline group presented indications of osteoporosis in the femoral joint, as evidenced by irregularities in the surrounding bone due to bone absorption and low bone density, particularly in the upper half of the femur where the bone nearly disappeared (Figure 6b). In the risedronate group (Figure 6c), administering total COL, COL < 1 kDa, COL: 1–10 kDa, or COL > 10 kDa (100 or 300 mg/kg/day) was shown to attenuate total cartilage destruction in a dose‐dependent manner, as evidenced by white cartilaginous mineral deposits in the trabecular bone of the proximal femur (Figure 6d–i). Moreover, the Mankin scores of OVX mice treated with total COL, COL < 1 kDa, COL: 1–10 kDa, or COL > 10 kDa (100 or 300 mg/kg/day) were significantly lower than the saline group (^#^ *p* < .05, ^##^ *p* < .01, and ^###^ *p* < .001, respectively) (Figure 6j).

### 3.8 Low‐MW collagen peptide elevated the density of femur bones in ovariectomized osteoporosis mice

**FIGURE 7:**
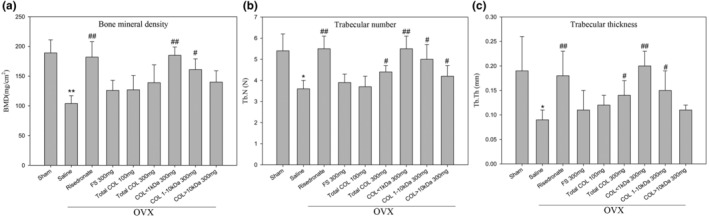
X‐ray analysis of femoral tissue from mice, including the sham group; negative control group (Saline); positive control group (Risedronate); FS (300 mg/kg/day); total COL (100 and 300 mg/kg/day); COL < 1 kDa (300 mg/kg/day); COL: 1–10 kDa (300 mg/kg/day); and COL > 10 kDa (300 mg/kg/day). Quantification of the (a) bone mineral density, (b) trabecular number, or (c) thickness of OVX mice in each group (*n* = 5). *p < .05, **p < .01, ^#^ *p* < .05, and ^##^ *p* < .01 indicate a significant difference compared with sham or saline groups, respectively.

We also evaluated that to compare femurs in terms of bone architecture and soft bone loss using the radiographic X‐ray in ovariectomized mice. Results show that the bone mineral density (Figure 7a), trabecular number (Figure 7b), or thickness (Figure 7c) of OVX mice in each group treated with total COL, COL < 1 kDa, COL: 1–10 kDa, or COL > 10 kDa (300 mg/kg/day) were significantly higher than the saline group (^#^ *p* < .05, ^##^ *p* < .01, and ^###^ *p* < .001, respectively).

## 4 DISCUSSION

Roughly one‐third of postmenopausal women are affected by osteoporosis, of whom approximately 50% experience bone fractures (Gosset et al., 2021). Postmenopausal osteoporosis refers to a state of estrogen deficiency characterized by low bone mass and bone fragility. Estrogen deficiency and age are important risk factors for the pathogenesis of osteoporosis (Nian et al., 2009). An imbalance between osteoclast and osteoblast activity shifts the balance away from bone formation toward bone resorption (Hertrampf et al., 2008). Existing therapies for osteoporosis focus on estrogen hormone replacement, aimed at maintaining bone density after menopause. Unfortunately, estrogen therapy increases the risk of breast cancer, heart attack, blood clots, and stroke. A preferred approach to maintaining the balance between bone formation and bone resorption is to control osteoblast and osteoclast activity. In the current study, we examined the effects of CP extracted from milkfish scales on RANKL‐induced osteoclast differentiation in RAW264.7 cells, osteoblast proliferation in MG‐63 cells, and osteoporosis in an animal model.

Previous studies have claimed that osteoporosis is caused by reversible collagen loss (Shuster, 2020). It appears that supplementing collagen hydrolysate is a safe approach to inducing the ERK/MAPK signaling pathway to promote alpha 1 (COLIA1) expression (Kim et al., 2013). Collagen peptides have been shown to promote bone formation (Watanabe‐Kamiyama et al., 2010). The type I collagen and hydroxyapatite extracted from milkfish scales in the current study are closely related to the materials found in human bone. Researchers have demonstrated that the fish collagen peptides are generally recognized as safe and have a low cytotoxicity profile (Chen et al., 2018; Zhu et al., 2014). As a waste from fish processing facilities, fish scales are abundantly available and inexpensive.

RAW264.7 cells are monocyte/macrophage‐like cells generated in a BALB/c mouse cell line infected with the Abelson leukemia virus (Taciak et al., 2018). Although RAW264.7 cells are not a homogeneous osteoclast cell line, they are useful as an osteoclast model due to their availability, ease of culturing, and sensitivity to RANKL stimulation (Collin‐Osdoby et al., 2003). Previous studies have documented various methods by which RAW264.7 cells can be used to study RANKL‐induced osteoclastogenic differentiation (Nguyen & Nohe, 2017; Sapkota et al., 2018; Song et al., 2019). MG‐63 cells were originally isolated from human osteosarcoma and have subsequently been used to evaluate the effects of implant materials on cellular environments (Lou et al., 2010).

The nuclear factor of activated T‐cell cytoplasmic (NFATc) is a transcription factor family regulated by calcium signals or auto‐amplification. It has been shown to play a pivotal role in regulating osteoclast differentiation. Tartrate‐resistant acid phosphatase (TRAP) has been linked to osteoclastic bone resorption. We observed significant decreases in the mRNA expression of these genes after treatment with COL peptides <1 kDa for 2 or 4 days (Figure 2). RANKL is produced by osteoblasts and affects osteoclast activation associated with bone absorption (An et al., 2016). A wide range of cell signals, hormones, and growth factors are regulated by RANKL as well as its two receptors, RANK and osteoprotegerin (OPG). RANKL binding to RANK promotes osteoclast differentiation and functionality. Risedronate prevents osteoporosis in postmenopausal women (Nuti, 2014). Excess OPG acts as a decoy receptor and competes with the conventional RANK receptor, thereby inhibiting the RANKL ligand from binding to RANK on osteoclasts (Boyce & Xing, 2008). Thus, the increased OPG mRNA expression level following treatment of MG‐63 cells with collagen peptide <1 kDa resulted in a deficit in RANKL signaling. We posit that the reduction in RANKL may be due to osteoclast apoptosis or impeded osteoclastogenesis (Figure 3d–f).

Further, ALP is widely used as a marker fo.r osteogenesis observable during cell proliferation and matrix maturation (days 10–15) (Feng et al., 2008). It is of note that alkaline phosphatase (ALP), found in bones, is produced by osteoblasts, the cells responsible for osteogenesis. It exists as a tetramer and is anchored to the plasma membrane of osteoblasts or attached to their matrix vesicles through a glycosylphosphatidylinositol (GPI) linkage at the enzyme's carboxyl‐terminal (Beck Jr. et al., 2000). Further, markers of bone formation are substances produced by active osteoblasts at various stages of their development. These markers are considered indicative of different aspects of osteoblast function and the process of bone formation (Shetty et al., 2016). In line with the above evidence and our in vitro OPG mRNA levels, the in vivo results demonstrated significantly elevated ALP levels (Figure 5b) along with improved bone density, particularly in the COL < 1 kDa (300 mg/kg/day) treatment group during histomorphometric analysis of femur bone (Figure 6g). Collectively, our results implied osteogenesis‐promoting effects of COL < 1 kDa in ovariectomy‐induced osteoporosis.

Previous research has shown that low‐molecular‐weight collagen peptide or fish collagen peptide is a potential candidate for the treatment of OA that stimulates cartilage regeneration using a rabbit anterior cruciate ligament transection (ACLT) model of induced OA and chondrocytes isolated from a patient with OA (Lee et al., 2021; Ohnishi et al., 2013). Fish collagen hydrolysates were also shown to enhance collagen production, viability, and proliferation in human articular chondrocytes (Bourdon et al., 2021). In addition, OVX mice or rats have previously been used in the study of osteoporosis following menopause (Lemini et al., 2015; Strom et al., 2012; Zhang et al., 2014). Ovariectomized mice have been shown to mimic the bone loss and clinical symptoms of postmenopausal osteoporosis induced by estrogen deficiency. Researchers have developed several agents aimed at preventing bone resorption, including bisphosphonates, selective estrogen receptor modulators, and RANK ligand inhibitors. Collagen peptide from deer sinew or Pacific cod (*Gadus macrocephalus*) has a protective effect against bone loss or anti‐osteoporosis in ovariectomized rats (Zhang et al., 2014; Zhang et al., 2018). In the current study, decreases in all histomorphometric indices pointed to a reduction in bone structure and mass, while increases in resorption indices in the OVX sham controls pointed to increases in bone turnover and decreases in bone formation, both of which are linked to estrogen‐deficient osteoporosis. Additionally, our results show that the collagen peptide of COL1 kDa treatment could reduce bone density loss in the mouse model of postmenopausal osteoporosis (Figure 6), which is consistent with previous findings (Lee et al., 2021).

Besides various positive outcomes, our study includes a limitation. Specifically, though we lacked alizarin red staining data, we concluded osteoblast differentiation solely based on enhanced ALP and OPG mRNA levels (Figure 3). Reportedly, ALP begins to produce and mature osteogenic extracellular matrix eventually expressing genes involved in extracellular matrix, mineralization like osteocalcin and osteopontin. This highly regulated gene expression and cellular differentiation program is regulated by the expression and activity of various transcription factors (Zhang, 2010). MS‐derived collagen peptides inhibited osteoclast differentiation in RAW264.7 cells following an insult with nuclear factor kappa‐B ligand (RANKL). It also enhanced osteoblast proliferation in MG‐63 cells, possibly through downregulating NFATc1 and TRAP (Figure 2) while upregulating ALP and OPG mRNA levels (Figure 3). Furthermore, COL1 kDa also inhibited bone density loss in osteoporotic mice, which may involve the function of osteogenic and osteoclastic (Figures 6 and 7). However, the H&E stain and Mankin's score all showed that COL 1 kDa prevented the degradation level of the articular cartilage, and COL 1 kDa also recovered the level of bone mineral density, trabecular number, and trabecular thickness. The above evidence all supports the benefit of COL 1 kDa in the treatment of osteoporosis. To confirm this hypothesis, more research will be required.

## 5 CONCLUSIONS

Taken together, collagen peptides may reduce RANKL‐induced osteoclast activity while promoting osteoblast synthesis, and therefore may be a potential therapeutic candidate for the prevention and control of osteoporosis.

## AUTHOR CONTRIBUTIONS

**Jiunn‐Jye Chuu:** Conceptualization (equal); data curation (equal); formal analysis (equal); funding acquisition (equal); project administration (equal); resources (equal); software (equal); supervision (equal); writing – original draft (equal); writing – review and editing (equal). **Jeng‐Wei Lu:** Conceptualization (equal); data curation (equal); formal analysis (equal); funding acquisition (equal); investigation (equal); methodology (equal); resources (equal); validation (equal); writing – review and editing (equal). **Hung‐Ju Chang:** Conceptualization (equal); data curation (equal); formal analysis (equal); investigation (equal); methodology (equal); validation (equal). **You‐Hsiang Chu:** Formal analysis (equal); investigation (equal); methodology (equal); software (equal); validation (equal). **Yi‐Jen Peng:** Formal analysis (equal); investigation (equal); resources (equal); validation (equal). **Yi‐Jung Ho:** Data curation (equal); investigation (equal); methodology (equal); resources (equal). **Pei‐Hung Shen:** Formal analysis (equal); investigation (equal); methodology (equal); resources (equal). **Yu‐Shuan Cheng:** Data curation (equal); formal analysis (equal); investigation (equal); methodology (equal); validation (equal). **Chia‐Hui Cheng:** Conceptualization (equal); data curation (equal); formal analysis (equal); investigation (equal); methodology (equal); software (equal); visualization (equal). **Yi‐Chien Liu:** Data curation (equal); investigation (equal); methodology (equal); validation (equal). **Chih‐Chien Wang:** Conceptualization (equal); data curation (equal); formal analysis (equal); investigation (equal); methodology (equal); project administration (equal); resources (equal); supervision (equal); validation (equal); visualization (equal); writing – original draft (equal); writing – review and editing (equal).

## CONFLICT OF INTEREST STATEMENT

The authors report no conflict of interest in relation to the work.

## Data Availability

The data that support the findings of this study are available on request from the corresponding author.
